# Supplementary material for: Modeling glioblastoma heterogeneity as a dynamic network of cell states
Source: Mol Syst Biol. 2021 Sep 16;17(9):e10105. doi: 10.15252/msb.202010105 (PMC8444284; doi:10.15252/msb.202010105)
Supplement: Supplementary file 5 — Source Data for Figure 3 [file MSB-17-e10105-s001.zip › Figure3A_sourcedata/GSEA_3065/nosigdb_state5.GseaPreranked.1622718435914/DOROSZKO_U3180MG_CLUSTER_2.html]

Details for gene set DOROSZKO\_U3180MG\_CLUSTER\_2[GSEA]

|  || Dataset | state5 |
| Phenotype | NoPhenotypeAvailable |
| Upregulated in class | na\_pos |
| GeneSet | DOROSZKO\_U3180MG\_CLUSTER\_2 |
| Enrichment Score (ES) | 0.6988746 |
| Normalized Enrichment Score (NES) | 3.3317251 |
| Nominal p-value | 0.0 |
| FDR q-value | 0.0 |
| FWER p-Value | 0.0 |
Table: GSEA Results Summary

  

Fig 1: Enrichment plot: DOROSZKO\_U3180MG\_CLUSTER\_2      
 Profile of the Running ES Score & Positions of GeneSet Members on the Rank Ordered List

  

| PROBE | GENE SYMBOL | GENE\_TITLE | RANK IN GENE LIST | RANK METRIC SCORE | RUNNING ES | CORE ENRICHMENT || 1 | SNTG1 |  |  | 4 | 0.988 | 0.0083 | Yes |
| 2 | VMP1 |  |  | 5 | 0.967 | 0.0169 | Yes |
| 3 | KCNQ1OT1 |  |  | 8 | 0.931 | 0.0249 | Yes |
| 4 | NOVA1 |  |  | 10 | 0.870 | 0.0326 | Yes |
| 5 | PTPRZ1 |  |  | 12 | 0.862 | 0.0401 | Yes |
| 6 | WSB1 |  |  | 13 | 0.861 | 0.0478 | Yes |
| 7 | CD9 |  |  | 14 | 0.857 | 0.0554 | Yes |
| 8 | NEAT1 |  |  | 17 | 0.830 | 0.0625 | Yes |
| 9 | MEG3 |  |  | 22 | 0.783 | 0.0690 | Yes |
| 10 | PDIA6 |  |  | 29 | 0.742 | 0.0749 | Yes |
| 11 | APLP2 |  |  | 31 | 0.733 | 0.0813 | Yes |
| 12 | HSP90B1 |  |  | 33 | 0.729 | 0.0877 | Yes |
| 13 | KANSL1 |  |  | 34 | 0.721 | 0.0941 | Yes |
| 14 | APP |  |  | 38 | 0.712 | 0.1001 | Yes |
| 15 | NKTR |  |  | 42 | 0.700 | 0.1059 | Yes |
| 16 | HSPA5 |  |  | 43 | 0.698 | 0.1121 | Yes |
| 17 | TNC |  |  | 45 | 0.680 | 0.1181 | Yes |
| 18 | RCN2 |  |  | 46 | 0.671 | 0.1240 | Yes |
| 19 | FUS |  |  | 47 | 0.665 | 0.1299 | Yes |
| 20 | SF1 |  |  | 48 | 0.655 | 0.1357 | Yes |
| 21 | PDIA3 |  |  | 49 | 0.652 | 0.1415 | Yes |
| 22 | ARGLU1 |  |  | 50 | 0.642 | 0.1472 | Yes |
| 23 | LAPTM4A |  |  | 51 | 0.641 | 0.1529 | Yes |
| 24 | CANX |  |  | 53 | 0.634 | 0.1585 | Yes |
| 25 | CALR |  |  | 55 | 0.631 | 0.1640 | Yes |
| 26 | LRPAP1 |  |  | 57 | 0.619 | 0.1693 | Yes |
| 27 | GPM6A |  |  | 60 | 0.609 | 0.1745 | Yes |
| 28 | B2M |  |  | 61 | 0.601 | 0.1799 | Yes |
| 29 | ATP6AP2 |  |  | 62 | 0.597 | 0.1852 | Yes |
| 30 | DDX17 |  |  | 64 | 0.595 | 0.1903 | Yes |
| 31 | LUC7L3 |  |  | 65 | 0.589 | 0.1956 | Yes |
| 32 | DDOST |  |  | 67 | 0.586 | 0.2007 | Yes |
| 33 | LAMP2 |  |  | 69 | 0.584 | 0.2057 | Yes |
| 34 | NCAM1 |  |  | 70 | 0.581 | 0.2109 | Yes |
| 35 | ADGRG1 |  |  | 74 | 0.567 | 0.2156 | Yes |
| 36 | DDR1 |  |  | 81 | 0.551 | 0.2198 | Yes |
| 37 | SLC1A3 |  |  | 82 | 0.550 | 0.2247 | Yes |
| 38 | ACSL3 |  |  | 88 | 0.543 | 0.2289 | Yes |
| 39 | LMAN1 |  |  | 95 | 0.533 | 0.2329 | Yes |
| 40 | TTC14 |  |  | 100 | 0.526 | 0.2371 | Yes |
| 41 | PRCP |  |  | 103 | 0.523 | 0.2416 | Yes |
| 42 | SEMA5A |  |  | 111 | 0.516 | 0.2453 | Yes |
| 43 | PDIA4 |  |  | 112 | 0.514 | 0.2499 | Yes |
| 44 | TMEM165 |  |  | 114 | 0.510 | 0.2543 | Yes |
| 45 | ITGAV |  |  | 118 | 0.508 | 0.2585 | Yes |
| 46 | ITGB8 |  |  | 126 | 0.503 | 0.2621 | Yes |
| 47 | BSG |  |  | 135 | 0.493 | 0.2656 | Yes |
| 48 | CCDC14 |  |  | 136 | 0.492 | 0.2699 | Yes |
| 49 | CCDC47 |  |  | 140 | 0.490 | 0.2739 | Yes |
| 50 | N4BP2L2 |  |  | 142 | 0.489 | 0.2782 | Yes |
| 51 | GUSB |  |  | 143 | 0.489 | 0.2825 | Yes |
| 52 | RRBP1 |  |  | 147 | 0.486 | 0.2865 | Yes |
| 53 | EFHC1 |  |  | 148 | 0.486 | 0.2908 | Yes |
| 54 | PNISR |  |  | 150 | 0.482 | 0.2950 | Yes |
| 55 | CLSTN1 |  |  | 154 | 0.478 | 0.2989 | Yes |
| 56 | PPIB |  |  | 156 | 0.477 | 0.3030 | Yes |
| 57 | ITGB1 |  |  | 157 | 0.477 | 0.3072 | Yes |
| 58 | DYNC1H1 |  |  | 160 | 0.475 | 0.3112 | Yes |
| 59 | DPP6 |  |  | 161 | 0.475 | 0.3154 | Yes |
| 60 | CLU |  |  | 162 | 0.474 | 0.3196 | Yes |
| 61 | GLG1 |  |  | 170 | 0.463 | 0.3229 | Yes |
| 62 | DLL3 |  |  | 171 | 0.463 | 0.3271 | Yes |
| 63 | SPG7 |  |  | 185 | 0.450 | 0.3295 | Yes |
| 64 | SLC16A1 |  |  | 186 | 0.449 | 0.3335 | Yes |
| 65 | PRKCSH |  |  | 189 | 0.447 | 0.3373 | Yes |
| 66 | VCAN |  |  | 191 | 0.444 | 0.3411 | Yes |
| 67 | PNN |  |  | 195 | 0.442 | 0.3447 | Yes |
| 68 | MAGI1 |  |  | 196 | 0.442 | 0.3486 | Yes |
| 69 | SELENOS |  |  | 197 | 0.441 | 0.3525 | Yes |
| 70 | RSRP1 |  |  | 200 | 0.439 | 0.3562 | Yes |
| 71 | HEPN1 |  |  | 203 | 0.437 | 0.3598 | Yes |
| 72 | ATF6B |  |  | 211 | 0.432 | 0.3629 | Yes |
| 73 | ATP2A2 |  |  | 212 | 0.431 | 0.3667 | Yes |
| 74 | MATN2 |  |  | 213 | 0.431 | 0.3705 | Yes |
| 75 | POR |  |  | 215 | 0.430 | 0.3742 | Yes |
| 76 | RCN1 |  |  | 217 | 0.427 | 0.3779 | Yes |
| 77 | RTN4 |  |  | 221 | 0.425 | 0.3813 | Yes |
| 78 | SLC3A2 |  |  | 222 | 0.424 | 0.3851 | Yes |
| 79 | SLC25A37 |  |  | 223 | 0.422 | 0.3888 | Yes |
| 80 | RDH11 |  |  | 224 | 0.422 | 0.3926 | Yes |
| 81 | MFSD10 |  |  | 226 | 0.421 | 0.3962 | Yes |
| 82 | PSAP |  |  | 227 | 0.420 | 0.4000 | Yes |
| 83 | TM9SF2 |  |  | 229 | 0.419 | 0.4036 | Yes |
| 84 | MFGE8 |  |  | 233 | 0.418 | 0.4069 | Yes |
| 85 | WDR73 |  |  | 236 | 0.414 | 0.4104 | Yes |
| 86 | PABPN1 |  |  | 237 | 0.414 | 0.4140 | Yes |
| 87 | FAM133B |  |  | 257 | 0.405 | 0.4154 | Yes |
| 88 | LRP1 |  |  | 259 | 0.404 | 0.4189 | Yes |
| 89 | HM13 |  |  | 260 | 0.404 | 0.4225 | Yes |
| 90 | CDH2 |  |  | 261 | 0.403 | 0.4261 | Yes |
| 91 | MUM1 |  |  | 263 | 0.401 | 0.4295 | Yes |
| 92 | SYT11 |  |  | 265 | 0.400 | 0.4329 | Yes |
| 93 | ACAP3 |  |  | 267 | 0.399 | 0.4364 | Yes |
| 94 | TAF1D |  |  | 268 | 0.399 | 0.4399 | Yes |
| 95 | DDRGK1 |  |  | 272 | 0.398 | 0.4431 | Yes |
| 96 | CHD9 |  |  | 275 | 0.396 | 0.4464 | Yes |
| 97 | CD164 |  |  | 279 | 0.394 | 0.4496 | Yes |
| 98 | TTC19 |  |  | 282 | 0.392 | 0.4528 | Yes |
| 99 | ATP6AP1 |  |  | 288 | 0.388 | 0.4557 | Yes |
| 100 | AIG1 |  |  | 290 | 0.388 | 0.4590 | Yes |
| 101 | CCNL1 |  |  | 300 | 0.382 | 0.4613 | Yes |
| 102 | LAMP1 |  |  | 304 | 0.380 | 0.4644 | Yes |
| 103 | SPTLC1 |  |  | 305 | 0.380 | 0.4677 | Yes |
| 104 | RIC3 |  |  | 308 | 0.378 | 0.4709 | Yes |
| 105 | DST |  |  | 313 | 0.376 | 0.4737 | Yes |
| 106 | CREB5 |  |  | 321 | 0.373 | 0.4762 | Yes |
| 107 | FKBP10 |  |  | 337 | 0.365 | 0.4777 | Yes |
| 108 | SFXN5 |  |  | 342 | 0.364 | 0.4805 | Yes |
| 109 | RBM39 |  |  | 353 | 0.360 | 0.4825 | Yes |
| 110 | PTX3 |  |  | 355 | 0.360 | 0.4856 | Yes |
| 111 | SETD5 |  |  | 360 | 0.358 | 0.4883 | Yes |
| 112 | P4HTM |  |  | 362 | 0.357 | 0.4913 | Yes |
| 113 | UNC50 |  |  | 364 | 0.356 | 0.4944 | Yes |
| 114 | NSD1 |  |  | 365 | 0.356 | 0.4976 | Yes |
| 115 | FJX1 |  |  | 371 | 0.352 | 0.5001 | Yes |
| 116 | RGS12 |  |  | 384 | 0.348 | 0.5018 | Yes |
| 117 | CTSD |  |  | 388 | 0.347 | 0.5045 | Yes |
| 118 | SPPL2A |  |  | 396 | 0.343 | 0.5068 | Yes |
| 119 | GON4L |  |  | 397 | 0.342 | 0.5098 | Yes |
| 120 | SERINC1 |  |  | 398 | 0.342 | 0.5128 | Yes |
| 121 | OS9 |  |  | 402 | 0.340 | 0.5155 | Yes |
| 122 | SEC61A1 |  |  | 406 | 0.338 | 0.5182 | Yes |
| 123 | FNIP1 |  |  | 409 | 0.337 | 0.5209 | Yes |
| 124 | ASPH |  |  | 413 | 0.335 | 0.5235 | Yes |
| 125 | TRA2A |  |  | 419 | 0.335 | 0.5259 | Yes |
| 126 | EPM2AIP1 |  |  | 420 | 0.335 | 0.5289 | Yes |
| 127 | ADAM9 |  |  | 421 | 0.334 | 0.5319 | Yes |
| 128 | CTSF |  |  | 438 | 0.329 | 0.5329 | Yes |
| 129 | AC020916.1 |  |  | 452 | 0.325 | 0.5343 | Yes |
| 130 | ATP6V0B |  |  | 455 | 0.324 | 0.5369 | Yes |
| 131 | SUMF2 |  |  | 459 | 0.323 | 0.5394 | Yes |
| 132 | SERPINH1 |  |  | 460 | 0.322 | 0.5423 | Yes |
| 133 | PODXL2 |  |  | 464 | 0.321 | 0.5448 | Yes |
| 134 | RAB6A |  |  | 479 | 0.318 | 0.5460 | Yes |
| 135 | SLC25A36 |  |  | 482 | 0.317 | 0.5486 | Yes |
| 136 | PCMT1 |  |  | 493 | 0.313 | 0.5502 | Yes |
| 137 | MSI2 |  |  | 496 | 0.313 | 0.5527 | Yes |
| 138 | OFD1 |  |  | 502 | 0.311 | 0.5549 | Yes |
| 139 | RFX4 |  |  | 509 | 0.308 | 0.5569 | Yes |
| 140 | ZNF397 |  |  | 529 | 0.303 | 0.5574 | Yes |
| 141 | COL6A1 |  |  | 536 | 0.301 | 0.5594 | Yes |
| 142 | FGFR1 |  |  | 545 | 0.299 | 0.5611 | Yes |
| 143 | SLC22A17 |  |  | 550 | 0.297 | 0.5633 | Yes |
| 144 | B4GAT1 |  |  | 558 | 0.295 | 0.5651 | Yes |
| 145 | SAR1B |  |  | 562 | 0.295 | 0.5673 | Yes |
| 146 | GATAD1 |  |  | 563 | 0.295 | 0.5700 | Yes |
| 147 | ZNF302 |  |  | 570 | 0.292 | 0.5719 | Yes |
| 148 | GTF2H2 |  |  | 579 | 0.290 | 0.5735 | Yes |
| 149 | NPDC1 |  |  | 581 | 0.290 | 0.5760 | Yes |
| 150 | BICD1 |  |  | 586 | 0.289 | 0.5781 | Yes |
| 151 | EIF2S3 |  |  | 590 | 0.288 | 0.5803 | Yes |
| 152 | EXOC4 |  |  | 592 | 0.287 | 0.5827 | Yes |
| 153 | SLC39A1 |  |  | 603 | 0.284 | 0.5840 | Yes |
| 154 | TNFRSF12A |  |  | 615 | 0.282 | 0.5853 | Yes |
| 155 | NSUN5 |  |  | 618 | 0.281 | 0.5875 | Yes |
| 156 | GRN |  |  | 626 | 0.279 | 0.5892 | Yes |
| 157 | GPATCH8 |  |  | 637 | 0.277 | 0.5905 | Yes |
| 158 | AUP1 |  |  | 640 | 0.277 | 0.5927 | Yes |
| 159 | M6PR |  |  | 641 | 0.277 | 0.5952 | Yes |
| 160 | RSBN1L |  |  | 650 | 0.275 | 0.5967 | Yes |
| 161 | PRKCA |  |  | 652 | 0.275 | 0.5990 | Yes |
| 162 | ZNF292 |  |  | 656 | 0.274 | 0.6011 | Yes |
| 163 | JPX |  |  | 657 | 0.274 | 0.6035 | Yes |
| 164 | MPZL1 |  |  | 666 | 0.272 | 0.6050 | Yes |
| 165 | NOC2L |  |  | 667 | 0.272 | 0.6074 | Yes |
| 166 | ZEB2 |  |  | 670 | 0.271 | 0.6096 | Yes |
| 167 | MTF2 |  |  | 677 | 0.269 | 0.6113 | Yes |
| 168 | SPECC1 |  |  | 689 | 0.266 | 0.6123 | Yes |
| 169 | ZBTB20 |  |  | 696 | 0.263 | 0.6140 | Yes |
| 170 | GOLIM4 |  |  | 699 | 0.262 | 0.6161 | Yes |
| 171 | PRPF38B |  |  | 714 | 0.259 | 0.6167 | Yes |
| 172 | SDC3 |  |  | 728 | 0.255 | 0.6175 | Yes |
| 173 | SREK1 |  |  | 729 | 0.255 | 0.6197 | Yes |
| 174 | ZNF207 |  |  | 732 | 0.254 | 0.6217 | Yes |
| 175 | HNRNPH1 |  |  | 736 | 0.253 | 0.6236 | Yes |
| 176 | JMJD1C |  |  | 749 | 0.251 | 0.6245 | Yes |
| 177 | ZNF638 |  |  | 753 | 0.250 | 0.6263 | Yes |
| 178 | RBM25 |  |  | 760 | 0.249 | 0.6278 | Yes |
| 179 | ST5 |  |  | 770 | 0.247 | 0.6290 | Yes |
| 180 | EXOC7 |  |  | 788 | 0.243 | 0.6292 | Yes |
| 181 | MAT2A |  |  | 794 | 0.242 | 0.6307 | Yes |
| 182 | STAT3 |  |  | 797 | 0.242 | 0.6326 | Yes |
| 183 | MACF1 |  |  | 805 | 0.240 | 0.6339 | Yes |
| 184 | MARK3 |  |  | 807 | 0.239 | 0.6360 | Yes |
| 185 | KTN1 |  |  | 820 | 0.238 | 0.6367 | Yes |
| 186 | CDK5RAP3 |  |  | 823 | 0.237 | 0.6385 | Yes |
| 187 | DDX5 |  |  | 827 | 0.236 | 0.6403 | Yes |
| 188 | PHF14 |  |  | 837 | 0.234 | 0.6413 | Yes |
| 189 | GPRC5B |  |  | 838 | 0.234 | 0.6434 | Yes |
| 190 | ADAR |  |  | 857 | 0.232 | 0.6433 | Yes |
| 191 | ZNF37A |  |  | 870 | 0.230 | 0.6440 | Yes |
| 192 | DHX36 |  |  | 871 | 0.230 | 0.6460 | Yes |
| 193 | WDR60 |  |  | 878 | 0.228 | 0.6473 | Yes |
| 194 | SLC12A9 |  |  | 881 | 0.228 | 0.6491 | Yes |
| 195 | RAB3IP |  |  | 883 | 0.228 | 0.6510 | Yes |
| 196 | GPBP1 |  |  | 891 | 0.227 | 0.6522 | Yes |
| 197 | TMUB2 |  |  | 892 | 0.227 | 0.6543 | Yes |
| 198 | CTSA |  |  | 897 | 0.226 | 0.6558 | Yes |
| 199 | CHD6 |  |  | 899 | 0.226 | 0.6577 | Yes |
| 200 | NEMF |  |  | 909 | 0.224 | 0.6586 | Yes |
| 201 | TBL1XR1 |  |  | 911 | 0.223 | 0.6605 | Yes |
| 202 | GPAA1 |  |  | 912 | 0.223 | 0.6625 | Yes |
| 203 | GPATCH2 |  |  | 918 | 0.222 | 0.6638 | Yes |
| 204 | WEE1 |  |  | 919 | 0.222 | 0.6658 | Yes |
| 205 | ZNF655 |  |  | 923 | 0.221 | 0.6674 | Yes |
| 206 | FAM53C |  |  | 932 | 0.220 | 0.6685 | Yes |
| 207 | SNRNP70 |  |  | 935 | 0.220 | 0.6702 | Yes |
| 208 | THOC2 |  |  | 954 | 0.217 | 0.6700 | Yes |
| 209 | NELFCD |  |  | 969 | 0.215 | 0.6703 | Yes |
| 210 | TMEM59L |  |  | 972 | 0.214 | 0.6719 | Yes |
| 211 | BAZ2B |  |  | 974 | 0.214 | 0.6737 | Yes |
| 212 | IFNGR1 |  |  | 983 | 0.213 | 0.6747 | Yes |
| 213 | ARFGAP2 |  |  | 988 | 0.212 | 0.6761 | Yes |
| 214 | TMEM87A |  |  | 999 | 0.211 | 0.6768 | Yes |
| 215 | AKAP9 |  |  | 1021 | 0.208 | 0.6762 | Yes |
| 216 | MKLN1 |  |  | 1028 | 0.207 | 0.6773 | Yes |
| 217 | CNOT4 |  |  | 1039 | 0.206 | 0.6780 | Yes |
| 218 | TRIM44 |  |  | 1074 | 0.202 | 0.6758 | Yes |
| 219 | TNRC6B |  |  | 1080 | 0.201 | 0.6770 | Yes |
| 220 | ANKRD12 |  |  | 1083 | 0.200 | 0.6785 | Yes |
| 221 | FSTL1 |  |  | 1086 | 0.200 | 0.6801 | Yes |
| 222 | SLC35F1 |  |  | 1092 | 0.199 | 0.6812 | Yes |
| 223 | TMEM30A |  |  | 1106 | 0.198 | 0.6815 | Yes |
| 224 | REV3L |  |  | 1140 | 0.193 | 0.6793 | Yes |
| 225 | RBM26 |  |  | 1154 | 0.191 | 0.6795 | Yes |
| 226 | ARHGEF2 |  |  | 1159 | 0.190 | 0.6807 | Yes |
| 227 | RNMT |  |  | 1163 | 0.190 | 0.6820 | Yes |
| 228 | CCDC66 |  |  | 1176 | 0.188 | 0.6823 | Yes |
| 229 | COPA |  |  | 1189 | 0.186 | 0.6825 | Yes |
| 230 | RNF170 |  |  | 1207 | 0.184 | 0.6822 | Yes |
| 231 | HEPACAM |  |  | 1214 | 0.183 | 0.6831 | Yes |
| 232 | NASP |  |  | 1225 | 0.182 | 0.6836 | Yes |
| 233 | PRRC2B |  |  | 1241 | 0.180 | 0.6834 | Yes |
| 234 | GOLGB1 |  |  | 1254 | 0.178 | 0.6836 | Yes |
| 235 | CCDC82 |  |  | 1258 | 0.178 | 0.6848 | Yes |
| 236 | SRSF10 |  |  | 1270 | 0.176 | 0.6851 | Yes |
| 237 | RAF1 |  |  | 1275 | 0.175 | 0.6862 | Yes |
| 238 | ZNF680 |  |  | 1282 | 0.174 | 0.6870 | Yes |
| 239 | DDX46 |  |  | 1284 | 0.174 | 0.6884 | Yes |
| 240 | ZNF800 |  |  | 1300 | 0.171 | 0.6882 | Yes |
| 241 | JAG1 |  |  | 1304 | 0.170 | 0.6894 | Yes |
| 242 | SPSB3 |  |  | 1327 | 0.169 | 0.6883 | Yes |
| 243 | CELF1 |  |  | 1335 | 0.168 | 0.6889 | Yes |
| 244 | NCSTN |  |  | 1348 | 0.166 | 0.6890 | Yes |
| 245 | SF3B1 |  |  | 1362 | 0.165 | 0.6890 | Yes |
| 246 | CTSL |  |  | 1384 | 0.163 | 0.6879 | Yes |
| 247 | TCF25 |  |  | 1396 | 0.162 | 0.6881 | Yes |
| 248 | TRMT13 |  |  | 1406 | 0.161 | 0.6885 | Yes |
| 249 | CDC37 |  |  | 1410 | 0.161 | 0.6895 | Yes |
| 250 | ZNF131 |  |  | 1414 | 0.160 | 0.6906 | Yes |
| 251 | PLPP3 |  |  | 1423 | 0.160 | 0.6911 | Yes |
| 252 | LFNG |  |  | 1442 | 0.158 | 0.6904 | Yes |
| 253 | HMGXB4 |  |  | 1444 | 0.158 | 0.6917 | Yes |
| 254 | KNOP1 |  |  | 1445 | 0.157 | 0.6931 | Yes |
| 255 | LONP2 |  |  | 1460 | 0.156 | 0.6928 | Yes |
| 256 | RNF115 |  |  | 1461 | 0.156 | 0.6942 | Yes |
| 257 | U2SURP |  |  | 1472 | 0.155 | 0.6944 | Yes |
| 258 | COMMD2 |  |  | 1487 | 0.154 | 0.6941 | Yes |
| 259 | PKN2 |  |  | 1491 | 0.153 | 0.6951 | Yes |
| 260 | UBR5 |  |  | 1500 | 0.152 | 0.6956 | Yes |
| 261 | SON |  |  | 1507 | 0.152 | 0.6962 | Yes |
| 262 | RIMKLB |  |  | 1517 | 0.151 | 0.6965 | Yes |
| 263 | CYHR1 |  |  | 1519 | 0.150 | 0.6977 | Yes |
| 264 | KMT2C |  |  | 1537 | 0.148 | 0.6970 | Yes |
| 265 | SRSF6 |  |  | 1558 | 0.146 | 0.6960 | Yes |
| 266 | XBP1 |  |  | 1564 | 0.145 | 0.6967 | Yes |
| 267 | SPAG9 |  |  | 1566 | 0.145 | 0.6979 | Yes |
| 268 | COLGALT2 |  |  | 1576 | 0.143 | 0.6981 | Yes |
| 269 | IP6K2 |  |  | 1621 | 0.140 | 0.6941 | Yes |
| 270 | IST1 |  |  | 1635 | 0.139 | 0.6939 | Yes |
| 271 | LUC7L |  |  | 1640 | 0.139 | 0.6946 | Yes |
| 272 | ARHGEF7 |  |  | 1647 | 0.138 | 0.6951 | Yes |
| 273 | DPF2 |  |  | 1648 | 0.138 | 0.6964 | Yes |
| 274 | CCDC88A |  |  | 1656 | 0.137 | 0.6968 | Yes |
| 275 | SPARCL1 |  |  | 1663 | 0.136 | 0.6973 | Yes |
| 276 | PBRM1 |  |  | 1675 | 0.135 | 0.6972 | Yes |
| 277 | EPN2 |  |  | 1688 | 0.134 | 0.6970 | Yes |
| 278 | ZRANB2 |  |  | 1693 | 0.134 | 0.6977 | Yes |
| 279 | DCLK2 |  |  | 1694 | 0.134 | 0.6989 | Yes |
| 280 | STX16 |  |  | 1718 | 0.132 | 0.6974 | No |
| 281 | NTM |  |  | 1729 | 0.131 | 0.6973 | No |
| 282 | SNRNP200 |  |  | 1747 | 0.130 | 0.6965 | No |
| 283 | NPAS3 |  |  | 1752 | 0.130 | 0.6972 | No |
| 284 | CLK1 |  |  | 1761 | 0.129 | 0.6974 | No |
| 285 | SACS |  |  | 1773 | 0.128 | 0.6972 | No |
| 286 | QTRT1 |  |  | 1795 | 0.127 | 0.6959 | No |
| 287 | CDC42BPA |  |  | 1817 | 0.125 | 0.6946 | No |
| 288 | ASH1L |  |  | 1827 | 0.125 | 0.6946 | No |
| 289 | CFLAR |  |  | 1828 | 0.125 | 0.6957 | No |
| 290 | RPAP2 |  |  | 1832 | 0.125 | 0.6965 | No |
| 291 | TIA1 |  |  | 1841 | 0.123 | 0.6966 | No |
| 292 | ABHD12 |  |  | 1843 | 0.123 | 0.6976 | No |
| 293 | CYR61 |  |  | 1850 | 0.123 | 0.6980 | No |
| 294 | PUS7L |  |  | 1854 | 0.122 | 0.6987 | No |
| 295 | KMT2A |  |  | 1876 | 0.120 | 0.6973 | No |
| 296 | SPTBN1 |  |  | 1880 | 0.120 | 0.6981 | No |
| 297 | GCC2 |  |  | 1949 | 0.115 | 0.6911 | No |
| 298 | PTCD3 |  |  | 1969 | 0.114 | 0.6899 | No |
| 299 | BDP1 |  |  | 1977 | 0.113 | 0.6901 | No |
| 300 | CWC25 |  |  | 1997 | 0.112 | 0.6888 | No |
| 301 | TCF4 |  |  | 2008 | 0.111 | 0.6886 | No |
| 302 | IGFBP3 |  |  | 2037 | 0.109 | 0.6863 | No |
| 303 | MCL1 |  |  | 2055 | 0.108 | 0.6853 | No |
| 304 | SRSF5 |  |  | 2060 | 0.108 | 0.6858 | No |
| 305 | ARHGAP21 |  |  | 2067 | 0.107 | 0.6860 | No |
| 306 | DAAM1 |  |  | 2069 | 0.107 | 0.6869 | No |
| 307 | IFRD1 |  |  | 2073 | 0.107 | 0.6875 | No |
| 308 | B3GAT3 |  |  | 2097 | 0.106 | 0.6857 | No |
| 309 | THAP5 |  |  | 2102 | 0.105 | 0.6862 | No |
| 310 | SMARCC2 |  |  | 2113 | 0.104 | 0.6859 | No |
| 311 | TERF1 |  |  | 2135 | 0.103 | 0.6844 | No |
| 312 | HNRNPU |  |  | 2204 | 0.098 | 0.6773 | No |
| 313 | MEIS2 |  |  | 2207 | 0.098 | 0.6779 | No |
| 314 | FASTK |  |  | 2243 | 0.095 | 0.6746 | No |
| 315 | TUFM |  |  | 2244 | 0.095 | 0.6755 | No |
| 316 | ESF1 |  |  | 2248 | 0.095 | 0.6760 | No |
| 317 | RPRD1A |  |  | 2265 | 0.094 | 0.6749 | No |
| 318 | RBM28 |  |  | 2271 | 0.094 | 0.6752 | No |
| 319 | CSNK2A1 |  |  | 2284 | 0.093 | 0.6746 | No |
| 320 | TRA2B |  |  | 2285 | 0.093 | 0.6754 | No |
| 321 | DDX18 |  |  | 2288 | 0.093 | 0.6760 | No |
| 322 | AFF4 |  |  | 2290 | 0.092 | 0.6767 | No |
| 323 | HES1 |  |  | 2302 | 0.092 | 0.6762 | No |
| 324 | CAND1 |  |  | 2307 | 0.091 | 0.6766 | No |
| 325 | SRSF1 |  |  | 2334 | 0.090 | 0.6743 | No |
| 326 | NOP56 |  |  | 2390 | 0.086 | 0.6686 | No |
| 327 | RBM4B |  |  | 2400 | 0.085 | 0.6683 | No |
| 328 | DNM1L |  |  | 2401 | 0.085 | 0.6691 | No |
| 329 | NCK1 |  |  | 2405 | 0.085 | 0.6695 | No |
| 330 | HOOK3 |  |  | 2474 | 0.081 | 0.6622 | No |
| 331 | GNPAT |  |  | 2476 | 0.081 | 0.6628 | No |
| 332 | CPEB4 |  |  | 2511 | 0.078 | 0.6595 | No |
| 333 | RBM23 |  |  | 2513 | 0.078 | 0.6601 | No |
| 334 | SPTAN1 |  |  | 2534 | 0.077 | 0.6585 | No |
| 335 | PBXIP1 |  |  | 2536 | 0.077 | 0.6590 | No |
| 336 | SFPQ |  |  | 2548 | 0.077 | 0.6584 | No |
| 337 | MYO6 |  |  | 2580 | 0.075 | 0.6554 | No |
| 338 | HECTD1 |  |  | 2587 | 0.074 | 0.6554 | No |
| 339 | ABI2 |  |  | 2588 | 0.074 | 0.6561 | No |
| 340 | USP34 |  |  | 2594 | 0.074 | 0.6561 | No |
| 341 | NIN |  |  | 2601 | 0.074 | 0.6561 | No |
| 342 | SECISBP2 |  |  | 2605 | 0.073 | 0.6564 | No |
| 343 | ARID4A |  |  | 2629 | 0.072 | 0.6543 | No |
| 344 | HERPUD1 |  |  | 2634 | 0.072 | 0.6545 | No |
| 345 | CRTC3 |  |  | 2637 | 0.072 | 0.6549 | No |
| 346 | ANAPC7 |  |  | 2667 | 0.070 | 0.6521 | No |
| 347 | DDX6 |  |  | 2672 | 0.070 | 0.6523 | No |
| 348 | DPF3 |  |  | 2714 | 0.067 | 0.6480 | No |
| 349 | SUPT20H |  |  | 2735 | 0.066 | 0.6463 | No |
| 350 | PTBP1 |  |  | 2754 | 0.065 | 0.6448 | No |
| 351 | DDX3X |  |  | 2755 | 0.065 | 0.6453 | No |
| 352 | LRP10 |  |  | 2782 | 0.064 | 0.6428 | No |
| 353 | PRPSAP1 |  |  | 2822 | 0.061 | 0.6388 | No |
| 354 | NAV1 |  |  | 2828 | 0.061 | 0.6388 | No |
| 355 | PCM1 |  |  | 2851 | 0.060 | 0.6367 | No |
| 356 | CCS |  |  | 2872 | 0.059 | 0.6349 | No |
| 357 | SYNE2 |  |  | 2881 | 0.058 | 0.6345 | No |
| 358 | VPS37B |  |  | 2904 | 0.058 | 0.6324 | No |
| 359 | SRSF2 |  |  | 2908 | 0.057 | 0.6326 | No |
| 360 | KMT2E |  |  | 2912 | 0.057 | 0.6327 | No |
| 361 | MYCBP2 |  |  | 2925 | 0.056 | 0.6318 | No |
| 362 | TLE4 |  |  | 2935 | 0.056 | 0.6313 | No |
| 363 | DMAP1 |  |  | 2968 | 0.054 | 0.6280 | No |
| 364 | HAGHL |  |  | 2980 | 0.053 | 0.6272 | No |
| 365 | PTK2 |  |  | 2987 | 0.052 | 0.6269 | No |
| 366 | PPP1R16A |  |  | 3059 | 0.049 | 0.6190 | No |
| 367 | PRPF4B |  |  | 3071 | 0.048 | 0.6182 | No |
| 368 | KMT5B |  |  | 3116 | 0.046 | 0.6134 | No |
| 369 | SRSF7 |  |  | 3126 | 0.046 | 0.6128 | No |
| 370 | DNAJC2 |  |  | 3148 | 0.044 | 0.6107 | No |
| 371 | DTNA |  |  | 3219 | 0.040 | 0.6028 | No |
| 372 | MED6 |  |  | 3223 | 0.040 | 0.6028 | No |
| 373 | GDAP1 |  |  | 3246 | 0.039 | 0.6006 | No |
| 374 | FOXN2 |  |  | 3403 | 0.032 | 0.5826 | No |
| 375 | TTC37 |  |  | 3405 | 0.031 | 0.5827 | No |
| 376 | CTNNB1 |  |  | 3417 | 0.031 | 0.5817 | No |
| 377 | MPHOSPH8 |  |  | 3478 | 0.028 | 0.5749 | No |
| 378 | EWSR1 |  |  | 3617 | 0.023 | 0.5589 | No |
| 379 | FAM172A |  |  | 3622 | 0.023 | 0.5587 | No |
| 380 | USP3 |  |  | 3650 | 0.022 | 0.5557 | No |
| 381 | SMCHD1 |  |  | 3743 | 0.018 | 0.5451 | No |
| 382 | PSMC6 |  |  | 3744 | 0.018 | 0.5452 | No |
| 383 | KIF1B |  |  | 3759 | 0.017 | 0.5437 | No |
| 384 | TUBGCP2 |  |  | 3852 | 0.014 | 0.5331 | No |
| 385 | TCERG1 |  |  | 3871 | 0.013 | 0.5311 | No |
| 386 | DHX30 |  |  | 3918 | 0.011 | 0.5258 | No |
| 387 | ATRX |  |  | 3933 | 0.010 | 0.5242 | No |
| 388 | RIF1 |  |  | 4001 | 0.007 | 0.5164 | No |
| 389 | SAT2 |  |  | 4006 | 0.007 | 0.5160 | No |
| 390 | CHMP4A |  |  | 4022 | 0.006 | 0.5143 | No |
| 391 | IDS |  |  | 4033 | 0.006 | 0.5132 | No |
| 392 | ELMSAN1 |  |  | 4074 | 0.004 | 0.5085 | No |
| 393 | MTA1 |  |  | 4076 | 0.004 | 0.5084 | No |
| 394 | NAA15 |  |  | 4130 | 0.002 | 0.5022 | No |
| 395 | OSBPL1A |  |  | 4148 | 0.001 | 0.5002 | No |
| 396 | DTX3 |  |  | 4150 | 0.001 | 0.5001 | No |
| 397 | CNTN1 |  |  | 4200 | -0.001 | 0.4944 | No |
| 398 | TIMM44 |  |  | 4269 | -0.005 | 0.4865 | No |
| 399 | PHF20 |  |  | 4316 | -0.006 | 0.4811 | No |
| 400 | ABHD5 |  |  | 4327 | -0.007 | 0.4800 | No |
| 401 | ORAI2 |  |  | 4428 | -0.011 | 0.4684 | No |
| 402 | MTRF1L |  |  | 4451 | -0.012 | 0.4659 | No |
| 403 | SAFB2 |  |  | 4528 | -0.015 | 0.4571 | No |
| 404 | MYC |  |  | 4656 | -0.020 | 0.4424 | No |
| 405 | SRSF11 |  |  | 4687 | -0.021 | 0.4391 | No |
| 406 | CALD1 |  |  | 4714 | -0.022 | 0.4362 | No |
| 407 | CNOT2 |  |  | 4761 | -0.024 | 0.4310 | No |
| 408 | KIF21A |  |  | 4843 | -0.027 | 0.4218 | No |
| 409 | TDG |  |  | 4915 | -0.030 | 0.4137 | No |
| 410 | ARIH2 |  |  | 4930 | -0.030 | 0.4123 | No |
| 411 | ARRDC3 |  |  | 4938 | -0.031 | 0.4118 | No |
| 412 | GTF2I |  |  | 4961 | -0.032 | 0.4095 | No |
| 413 | TMEM120A |  |  | 5081 | -0.036 | 0.3958 | No |
| 414 | ANK2 |  |  | 5089 | -0.037 | 0.3953 | No |
| 415 | CTTN |  |  | 5123 | -0.038 | 0.3918 | No |
| 416 | PSRC1 |  |  | 5137 | -0.038 | 0.3906 | No |
| 417 | DDB1 |  |  | 5186 | -0.040 | 0.3853 | No |
| 418 | ANAPC5 |  |  | 5194 | -0.040 | 0.3849 | No |
| 419 | TAOK3 |  |  | 5204 | -0.041 | 0.3842 | No |
| 420 | YME1L1 |  |  | 5251 | -0.042 | 0.3792 | No |
| 421 | USP16 |  |  | 5327 | -0.045 | 0.3708 | No |
| 422 | DDIT3 |  |  | 5335 | -0.046 | 0.3703 | No |
| 423 | ATXN7L3B |  |  | 5403 | -0.049 | 0.3629 | No |
| 424 | USP33 |  |  | 5582 | -0.057 | 0.3425 | No |
| 425 | NCOR1 |  |  | 5617 | -0.059 | 0.3391 | No |
| 426 | CAMK2D |  |  | 5690 | -0.062 | 0.3312 | No |
| 427 | ARHGAP5 |  |  | 5716 | -0.063 | 0.3288 | No |
| 428 | AHCYL1 |  |  | 5817 | -0.066 | 0.3176 | No |
| 429 | FERMT1 |  |  | 5855 | -0.068 | 0.3139 | No |
| 430 | ABCE1 |  |  | 6018 | -0.075 | 0.2956 | No |
| 431 | ACADVL |  |  | 6028 | -0.076 | 0.2952 | No |
| 432 | IRS2 |  |  | 6033 | -0.076 | 0.2954 | No |
| 433 | TAOK1 |  |  | 6046 | -0.077 | 0.2946 | No |
| 434 | DNPEP |  |  | 6075 | -0.078 | 0.2921 | No |
| 435 | MLF1 |  |  | 6207 | -0.085 | 0.2774 | No |
| 436 | PGP |  |  | 6209 | -0.086 | 0.2781 | No |
| 437 | CHD1 |  |  | 6213 | -0.086 | 0.2785 | No |
| 438 | FOXO3 |  |  | 6238 | -0.087 | 0.2765 | No |
| 439 | LARS |  |  | 6288 | -0.090 | 0.2715 | No |
| 440 | CEBPZOS |  |  | 6289 | -0.090 | 0.2723 | No |
| 441 | LIMA1 |  |  | 6339 | -0.092 | 0.2674 | No |
| 442 | IFRD2 |  |  | 6353 | -0.093 | 0.2667 | No |
| 443 | TLK1 |  |  | 6386 | -0.094 | 0.2637 | No |
| 444 | SNHG9 |  |  | 6410 | -0.096 | 0.2619 | No |
| 445 | HSPA1B |  |  | 6593 | -0.108 | 0.2415 | No |
| 446 | DDX42 |  |  | 6768 | -0.119 | 0.2221 | No |
| 447 | PJA2 |  |  | 6820 | -0.122 | 0.2172 | No |
| 448 | HSPH1 |  |  | 6836 | -0.124 | 0.2166 | No |
| 449 | PRRC2A |  |  | 6952 | -0.133 | 0.2043 | No |
| 450 | RB1CC1 |  |  | 7156 | -0.151 | 0.1818 | No |
| 451 | IAH1 |  |  | 7325 | -0.170 | 0.1636 | No |
| 452 | CEBPZ |  |  | 7404 | -0.181 | 0.1561 | No |
| 453 | EMD |  |  | 7510 | -0.192 | 0.1454 | No |
| 454 | BCLAF1 |  |  | 7518 | -0.193 | 0.1463 | No |
| 455 | FAM120A |  |  | 7521 | -0.193 | 0.1478 | No |
| 456 | GNL3 |  |  | 7548 | -0.197 | 0.1465 | No |
| 457 | GADD45A |  |  | 7633 | -0.208 | 0.1385 | No |
| 458 | HSPA4 |  |  | 7791 | -0.232 | 0.1221 | No |
| 459 | SAT1 |  |  | 8162 | -0.314 | 0.0815 | No |
| 460 | PPP1CB |  |  | 8174 | -0.318 | 0.0831 | No |
| 461 | HSPD1 |  |  | 8418 | -0.393 | 0.0580 | No |
| 462 | LMNA |  |  | 8478 | -0.423 | 0.0549 | No |
| 463 | YBX3 |  |  | 8604 | -0.493 | 0.0446 | No |
Table: GSEA details [plain text format]

  

Fig 2: DOROSZKO\_U3180MG\_CLUSTER\_2: Random ES distribution      
 Gene set null distribution of ES for **DOROSZKO\_U3180MG\_CLUSTER\_2**
